# Identification of Diverse Bacteriophages Associated with Bees and Hoverflies

**DOI:** 10.3390/v17020201

**Published:** 2025-01-30

**Authors:** Rohan A. Bandoo, Simona Kraberger, Cahit Ozturk, Michael C. Lund, Qiyun Zhu, Chelsea Cook, Brian Smith, Arvind Varsani

**Affiliations:** 1 School of Life Sciences, Arizona State University, Tempe, AZ 85287, USA; rbandoo@asu.edu (R.A.B.); cahit.ozturk@asu.edu (C.O.); qiyun.zhu@asu.edu (Q.Z.); brianhsmith@asu.edu (B.S.); 2 The Biodesign Center for Fundamental and Applied Microbiomics, Arizona State University, Tempe, AZ 85287, USA; mclund2@asu.edu; 3 Department of Biological Sciences, Marquette University, Milwaukee, WI 53233, USA; chelsea.cook@marquette.edu; 4 Center for Evolution and Medicine, Arizona State University, Tempe, AZ 85287, USA; 5 Structural Biology Research Unit, Department of Integrative Biomedical Sciences, University of Cape Town, Rondebosch, Cape Town 7700, South Africa

**Keywords:** *Microviridae*, *Inoviridae*, *Caudovirictes*, honeybees, hoverflies, Nomia solitary bees

## Abstract

Bacteriophages are the most numerous, ubiquitous, and diverse biological entities on the planet. Prior studies have identified bacteriophages associated with pathogenic and commensal microbiota of honeybees. In this study we expand on what is known about bacteriophages from the lineages *Caudoviricetes*, *Inoviridae*, and *Microviridae*, which are associated with honeybees (Apidae, *Apis mellifera*), solitary bees of the genus *Nomia* (Halictidae, *Nomia*), and hoverflies (Syrphidae). The complete genomes of seven caudoviruses, seven inoviruses, and 288 microviruses were assembled from honeybees (*n* = 286) and hoverflies in Arizona (*n* = 2). We used bacterial host predictive software and sequence read mapping programs to infer the commensal and transient bacterial hosts of pollinating insects. Lastly, this study explores the phylogenetic relationships of microviruses sampled from bees, opportunistically sampled pollinating insects such as hoverflies, and blackflies.

## 1. Introduction

Bacteriophages (phages) are viruses that infect bacteria and are the most numerous biological entities on earth [1]. The proliferation of phages is associated with the development of bacterial communities [2] of the environment and host organisms. Phages can alter bacterial communities through phage–bacteria interactions such as the genetic transduction of antibiotic resistance genes [3,4], the maintenance of carbohydrate metabolism [5], the transduction of bacterial genes that may alter the phenotype [3,4,6], and the lysis of bacteria including pathogenic bacteria [7]. Changes in bacterial communities can affect the health of the associated host organisms that house the microbiome. These interactions continue to be explored as more bacteria and phages continue to be identified through microbial culturing and/or metagenomic workflows. Additionally, classifying phages has been challenging due to their high diversity both at a morphological and genomic level. While phages are highly diverse and ubiquitous biological entities, very little information is available on those associated with insects.

Honeybees, solitary bees, and hoverflies all play important roles in pollinating plants [8,9]. Honeybees in particular have been managed for centuries due to the prominent role they play in pollinating agriculturally important crops [10]. Solitary bees of the genus *Nomia* (Halictidae, *Nomia*) are semi-social ground-nesting alkali bees. These bees are relevant pollinators of plants such as alfalfa. Hoverflies or syrphids (family Syrphidae), in addition to being important incidental pollinators, produce larvae that feed on aphids, thrips, and other plant-feeding insects, making them important biological controls [11]. Over the last few decades, it has become evident that these pollinators are facing threats to their health [12]. This concern has led to increased research activity, in honeybees specifically, with an aim to understand factors impacting their health. As a result, phages in the class *Caudoviricetes* and the families *Cystoviridae*, *Inoviridae*, and *Microviridae* have been identified in pollinating insects [13,14,15,16,17,18]. Additionally, phages (and prophages) of the honeybee pathogens *Paenibacillus larvae* and *Melissococus plutonius,* the causative agents of American foulbrood disease and European foulbrood disease, respectively, have been identified [5]. Bueren et al. [5] suggest that prophages of the honeybee microbiome are highly specific to their host and can encode beneficial genes such as those that aid in carbohydrate metabolism. Bueren et al. [5] suggest that *Paenibacillus larvae* contain more prophage sequence regions than *Melissicoccus plutonius* and other core members of the honeybee gut microbiome.

Applications of phage therapeutics for the treatment of bacteria-induced foulbrood disease are concepts of growing intrigue. The identification of integrated prophages against pathogenic bacteria can have therapeutic applications if these phages can return to lytic replication cycles. A study by Busby et al. (2022) [13] to determine the global composition of bacteriophages (primarily caudoviruses and a few microviruses) associated with honeybees found that the majority of the bacteriophages are unique to the local region where the bees live. Furthermore, a study [13] identified bacteriophage sequence clusters in samples from the USA, Belgium, and Switzerland to be relatively more similar. A study focused on small DNA viruses in honeybees identified 70 microviruses within the *Gokushovirinae* subfamily [15]. While there are few studies that investigate the bacteriophages associated with honeybees, there appears to be no work documenting bacteriophages associated with hoverflies (syrphids) or *Nomia* solitary bees.

In this study, we identify the complete genomes of 288 microviruses, seven inoviruses and seven caudoviruses from honeybees, *Nomia* solitary bees, and hoverflies. Foraging honeybees were sampled from six locations within Arizona (USA), a location in Wisconsin (USA), and two apiaries in Westmoreland, Jamaica. The hoverflies and *Nomia* solitary bees were opportunistically sampled in Arizona (USA).

## 2. Materials and Methods

### 2.1. Sample Collection

**Figure 1:**
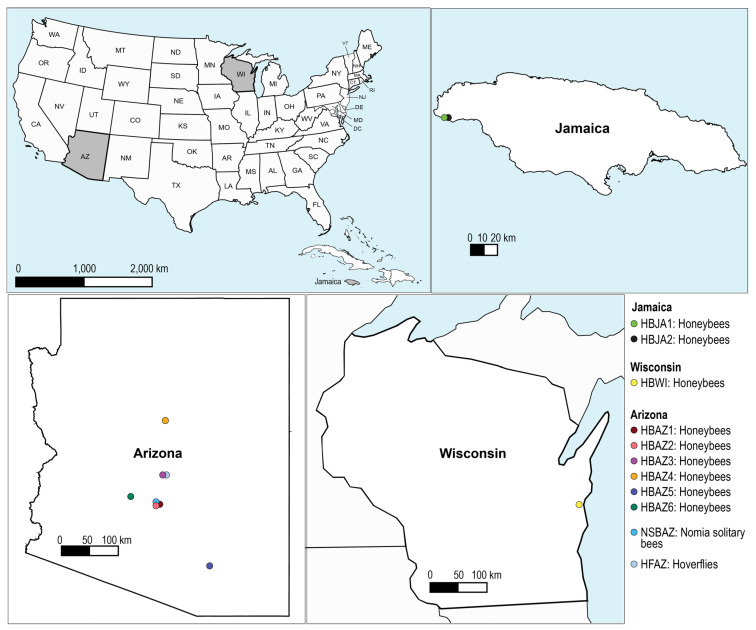
Map showing sampling sites for honeybees, solitary bees, and hoverflies for this study. The top left panel shows all three sampling regions in a larger map of North America and the Caribbean. The top right panel shows an enlargement of the Caribbean Island of Jamaica. The bottom two panels show enlargements of Arizona and Wisconsin in the USA. Sampling sites are displayed by colored circles based on insect type and location sampled.

Honeybees were collected from nine locations: Arizona State University (Tempe campus) in October 2022, Tempe Grand Canal in May 2023, Rackensack Canyon in April 2023, Sedona in September 2023, Tucson in October of 2023, and the White Tank Mountains in October of 2023, all in Arizona; Marquette University (Milwaukee, Wisconsin) in October 2023; and two separate locations in Westmoreland, Jamaica, in March 2023. Hoverflies were opportunistically collected from Rackensack Canyon in Arizona in April 2023. Lastly, solitary bees of the genus *Nomia* were opportunistically collected from Tempe Grand Canal in May 2023 (Figure 1). At each location, a minimum of 15 insects were collected.

### 2.2. Viral DNA Extraction

Individual insects were homogenized using pestles with 800 µL of SM Buffer (0.1 M NaCl, 10 mM MgSO_4_, 50 mM Tris-HCl pH 7.4) in 1.5 mL tubes. The homogenates were centrifuged at 8000× *g* rpm for 5 min, and 200 µL of the supernatant was used for DNA extraction using the High Pure Viral Nucleic Acid kit (Roche Diagnostic, Indianapolis, IN, USA).

### 2.3. High-Throughput Sequencing

Equal aliquots of the extracted viral DNA from individual insects from the eight locations were pooled per location. The pooled DNA was then used as a template for rolling circle amplification (RCA) using the TempliPhi 2000 kit (Thermo Fisher Scientific, Waltham, MA, USA). RCA products were then combined with the viral DNA at a 1:1 ratio and used to generate Illumina sequencing libraries using the Illumina DNA library prep kit (Illumina, San Diego, CA, USA). These libraries were sequenced on an Illumina NovaSeq X plus (Illumina, USA) sequencing platform at Psomagen (USA).

### 2.4. De Novo Assembly and Identification of Phage Genomes

Raw reads were quality-checked and trimmed with Trimmomatic v0.39 [19]. The trimmed reads were de novo assembled into contigs using MEGAHIT v1.2.0 [20]. Assembled contigs >1000 nts were analyzed against a viral RefSeq protein database (Release 220) using DIAMOND BLASTx [21]. Bacteriophage-like contigs were annotated using a combination of tools including Cenote-taker 2 [22], VIBRANT [23], and PHOLD [24,25,26,27,28]. The annotated genomes were manually checked using Geneious Prime 2023.2.1 (Dotmatics, Boston, MA, USA). Contigs with terminal redundancies were deemed circular and complete genomes.

Genome sequences belonging to the class *Caudoviricetes* and the families *Inoviridae* and *Microviridae* were identified. The genomes were oriented to the same starting point for each group. Each dataset was clustered at a 98% threshold using CD-Hit [29,30] to create vOTUs for the purpose of this study. Thus, sequences with >98% similarity within a group were treated as the same sequence under a vOTU grouping.

### 2.5. Read Mapping

To determine the presence/absence of the vOTUS across the various pools of bee and hoverfly samples, we used BBmap [31] to map the raw reads from the libraries to each individual vOTU.

### 2.6. Circo-Plot

An information aesthetic program called CIRCOS was used to illustrate the presence of viruses in common between sequencing pools [32].

### 2.7. Intergenomic Similarities

Intergenomic similarity matrices were generated using VIRIDIC [33] for caudoviruses and inoviruses.

### 2.8. Phage Putative Host Prediction

Prokaryotic hosts of the phages were predicted using iPHoP [34].

### 2.9. Phylogenetic Analyses

ViPTree [35] was used to infer proteomic phylogenetic trees for viruses in the lineages *Caudoviricetes*, *Inoviridae*, and *Microviridae*, independently. For the microviruses, we used ~6500 genomes available in GenBank with representatives of microviruses from the subfamily Bullavirinae for the ViPTree analysis. In the case of microviruses, the ViPTree was visualized and annotated in iTOLv6 [36].

### 2.10. Clustering of Microviruses

We used vConTACT 2 0.11.3 [37,38] to cluster the 288 microvirus sequences with sequences from GenBank (*n* = 6530) with the following parameters: –db ProkaryoticViralRefSeq211database –pc-evalue 0.1 –reported-alignments 100 –pc-inflation 1.2 –min-density 0 –vc-penalty 0 –vc-haircut 0.2. The resulting networks were visualized using Cytoscape 3.10.0 [39], which was also used to visualize and tag the clusters building on the work from [40] in terms of grouping.

## 3. Results and Discussion

To investigate the DNA bacteriophages associated with bees and hoverflies, we collected and processed individual insects, which were then pooled based on the type of insect and site. Nine of these pools are honeybees sampled in six locations within Arizona (sample ID # HBAZ1-6), one from Wisconsin (sample ID # HBWI), and two from Jamaica (sample ID # HBJA1-2) (Figure 1). Two pools are *Nomia* solitary bees (sample ID # NSBAZ) and hoverflies (sample ID # HFAZ), which were collected opportunistically from the Tempe Grand Canal, Tempe, AZ, USA and Rackensack Canyon, AZ, USA, respectively, pooled (Figure 1).

In total, we identified 302 bacteriophage genomes in the class *Caudoviricetes* (*n* = 7) and the families *Inoviridae* (*n* = 7) and *Microviridae* (*n* = 288) from 11 insect sample pools (Supplementary Materials, Table S1). The genome accession numbers and BioProject, BioSample, and SRA details, coupled with genome size and GC content, are provided in the Supplementary Materials (Table S1). The presence/absence and number of assembled viral genomes identified in the pools are summarized in Table 1. The number of bacteriophage genomes identified to be present in each pool ranged from 2 to 164. The DNA pool with the largest number of identified bacteriophages came from foraging honeybees sampled on the ASU campus in Arizona (sample ID # HBAZ1, *n* = 164). In descending order, the number of bacteriophage genomes identified by pool is as follows: ASU campus honeybees (sample ID # HBAZ1, *n* = 164), Tucson honeybees (sample ID # HBAZ5, *n* = 52), White Tank Mountain honeybees (sample ID # HBAZ6, *n* = 40), Rackensack Canyon honeybees (sample ID # HBAZ3, *n* = 35), Tempe Grand Canal honeybees (sample ID # HBAZ2, *n* = 13), Sedona honeybees (sample ID # HBAZ4, *n* = 7), Wisconsin honeybees (sample ID # HBWI, *n* = 6), honeybees from Jamaican apiary number one (sample ID # HBJA1, *n* = 2), honeybees from Jamaican apiary number two (sample ID # HBJA2, *n* = 2), Tempe Grand Canal *Nomia* solitary bees (sample ID # NSBAZ, *n* = 2), and Rackensack Canyon hoverflies (sample ID # HFAZ, *n* = 2).

### 3.1. Caudoviruses

Bacteriophages of the class *Caudoviricetes* are the most common taxonomic group of viruses. According to the 2024 ICTV viral metadata resource (https://ictv.global/vmr (accessed on 01 September 2024) [41], the class *Caudoviricetes*, has seven orders, 74 families, 121 subfamilies, 1498 genera, and 4840 species. The genome size of these bacteriophages is highly variable, ranging from ~18 kb to ~660 kb [42,43,44], with the most commonly identified conserved genes being those that code for the terminase, portal, and major capsid proteins [45].

**Figure 2:**
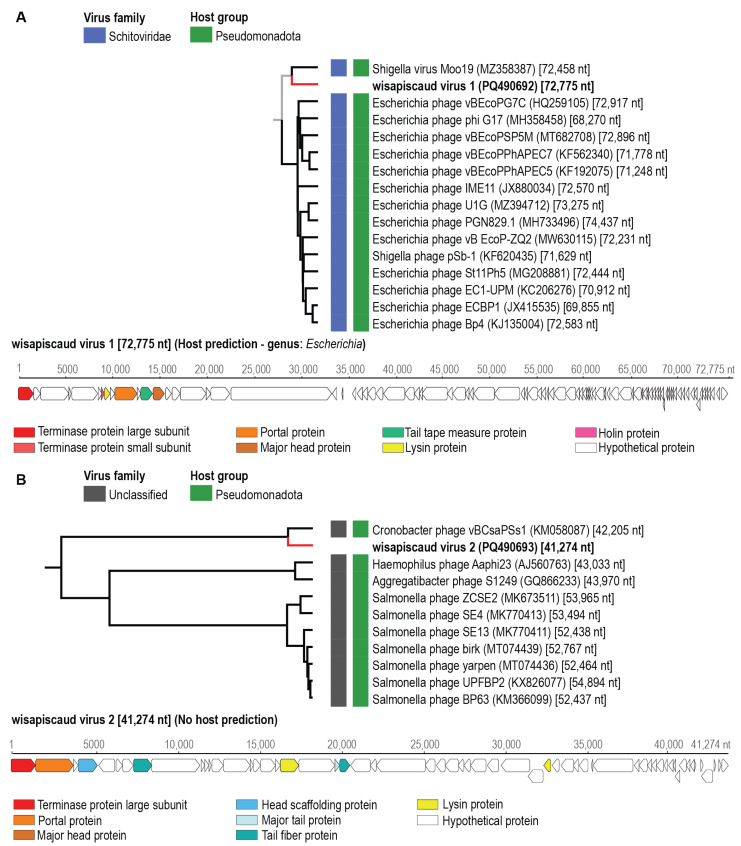
Individual ViPTree clusters depicting the proteomic relationships of (**A**) wisapiscaud virus 1 (PQ490692) and (**B**) wisapiscaud virus 2 (PQ490692) together with representative sequences from the class *Caudoviricetes.* The virus and putative hosts are shown next to the tree. The linearized genomes with their open reading frames are shown with some of the conserved protein-coding genes highlighted in different colors. Those in bold represent genome sequences from this study.

**Figure 3:**
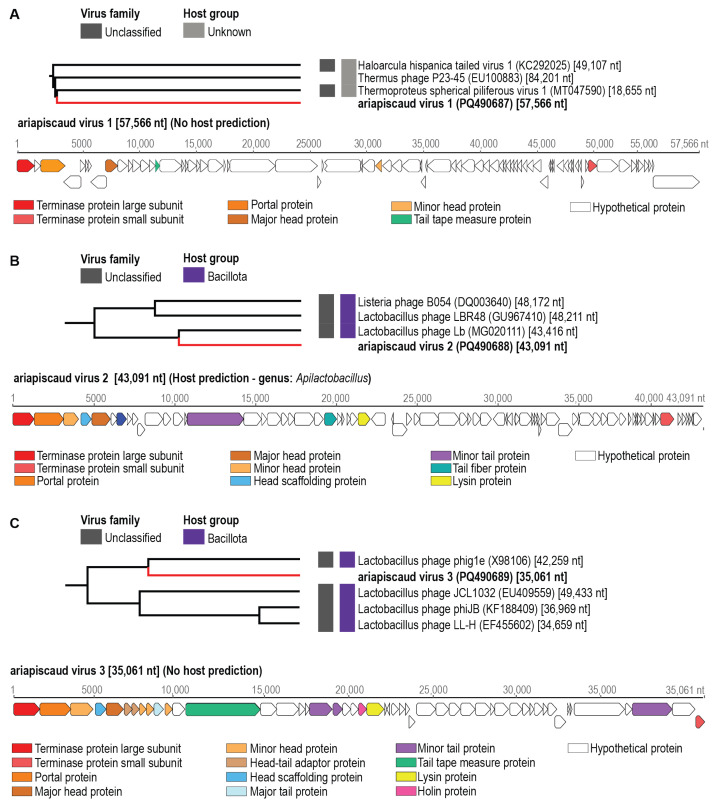
Individual ViPTree clusters depicting the proteomic relationships of (**A**) ariapiscaud virus 1 (PQ490687), (**B**) ariapiscaud virus 2 (PQ490688), and (**C**) ariapiscaud virus 3 (PQ490689) together with representative sequences from the class *Caudoviricetes.* The virus and putative hosts are shown next to the tree. The linearized genomes with their open reading frames are shown with some of the conserved protein-coding genes highlighted in different colors. Those in bold represent genome sequences from this study.

**Figure 4:**
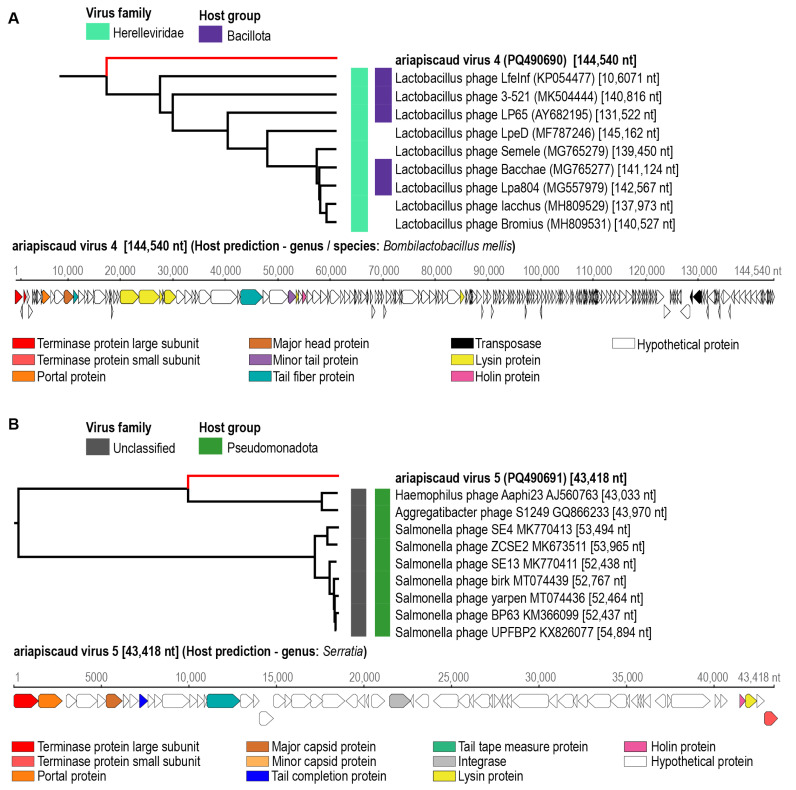
Individual ViPTree clusters depicting the proteomic relationships of (**A**) ariapiscaud virus 4 (PQ490690) and (**B**) ariapiscaud virus 5 (PQ490691) together with representative sequences from the class *Caudoviricetes.* The virus and putative hosts are shown next to the tree. The linearized genomes with their open reading frames are shown with some of the conserved protein-coding genes highlighted in different colors. Those in bold represent genome sequences from this study.

**Figure 5:**
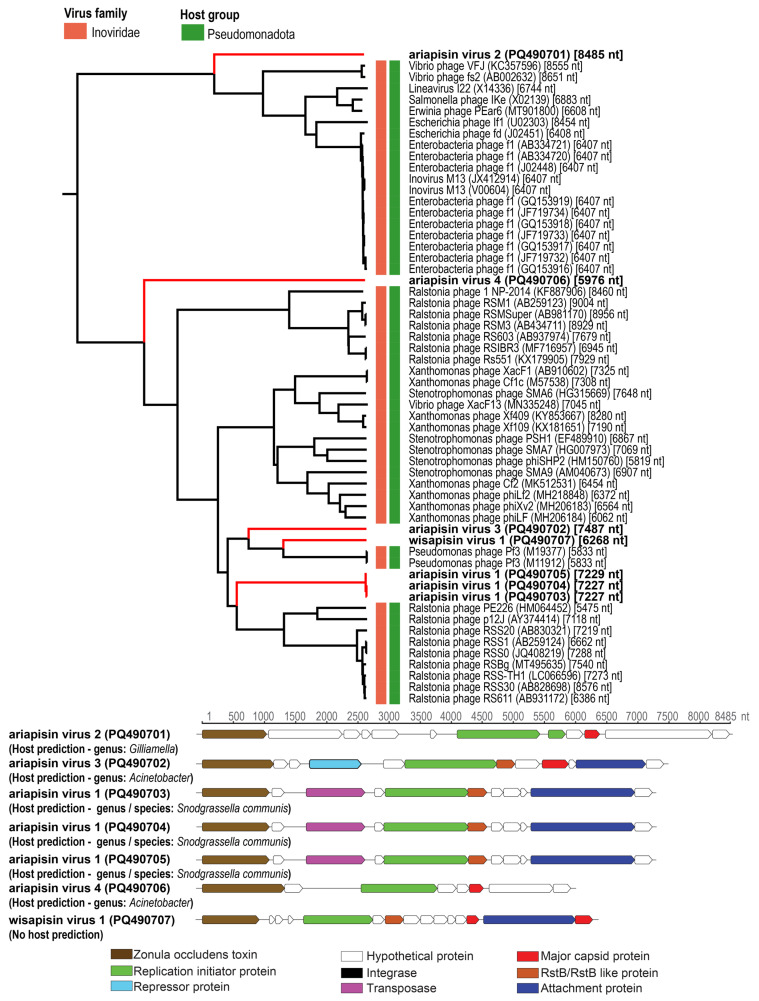
A ViPTre depicting the relationship of representative sequences of the family *Inoviridae* with the assembled inovirus genomes from this study. The putative virus and hosts are shown next to the tree. The linearized genomes with their open reading frames are shown with some of the conserved protein-coding genes highlighted in different colors. Those in bold represent genome sequences from this study.

**Figure 6:**
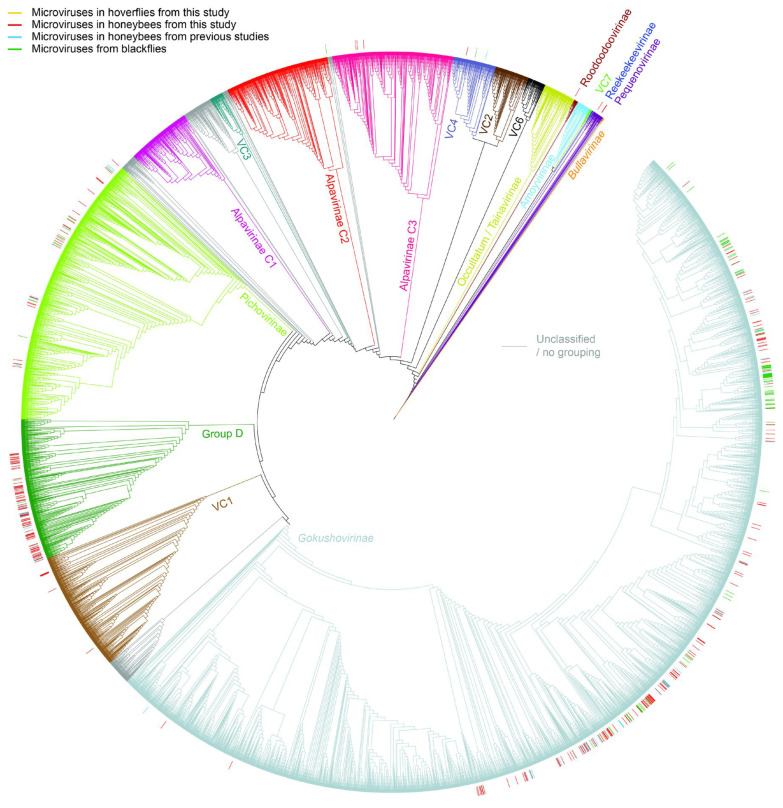
A circular ViPTree visualized in ITOL with the putative grouping determined using vConTACT 2. The branches of the tree are annotated by colors representing the groupings. Honeybee and hoverfly microvirus genomes from this study along with those previously identified from honeybees [15] and blackflies [61] are highlighted in a color strip around the tree.

**Figure 7:**
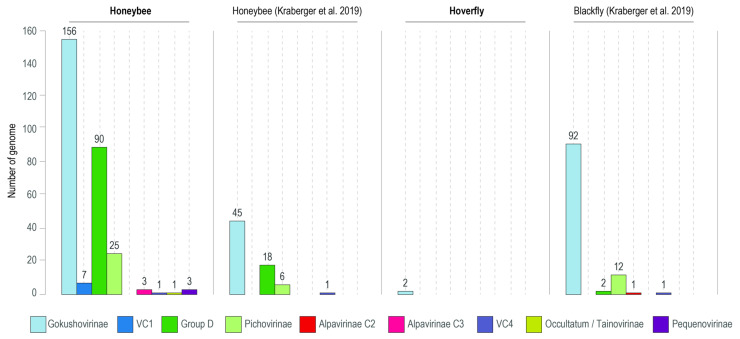
Summary of the microvirus genomes identified in this study from honeybees and hoverflies as well as those from honeybees by Kraberger et al. (2019) [15] and blackflies by Kraberger et al. (2019) [61], based on vConTACT 2 and ViPTree clustering.

Seven circular genomes ranging in size from ~35–145 kb encoding a large terminase, portal, capsid/head proteins, tail proteins, lysins/holins, and scaffold protein genes were identified from honeybees within the USA. The genomes were de novo assembled, identified, and annotated. These have been named ariapiscaud virus 1–5 (name derived from **Ari**zona **Apis caud**ovirus) and wisapiscaud virus 1–2 (name derived from **Wis**consin **Apis caud**ovirus) (Supplementary Materials, Table S1). The genome sequences of viruses ariapiscaud virus 1 (PQ490687, 57,566 nts), ariapiscaud virus 2 (PQ490688, 43,091 nts), ariapiscaud virus 3 (PQ490689, 35,061 nts), ariapiscaud virus 4 (PQ490690, 144,540 nts), ariapiscaud virus 5 (PQ490691, 43,418 nts), wisapiscaud virus 1 (PQ490692, 72,755 nts), and wisapiscaud virus 2 (PQ490693, 41,274 nts) were analyzed using VipTree to determine their proteomic phylogenetic clustering (Figure 2, Figure 3 and Figure 4). Turner et al (2021) [46] established a 95% genome-wide pairwise identity threshold for a species for bacteriophages. Ariapiscaud virus 1–5 and wisapiscaud virus 1–2 are all unique, each representing an individual virus species, i.e., sharing <95% genome-wide pairwise identity (Supplementary Materials, Figures S1–S7). Wisapiscaud virus 1 (PQ490692) shares 70.3% genome-wide identity (Supplementary Materials, Figure S1) with Shigella virus Moo19 (MZ358387) from a water pool in a bovine pasture, and wisapiscaud virus 2 (PQ490693) shares 70.7% genome-wide identity (Supplementary Materials, Figure S2) with Cronobacter phage vB_CsaP_Ss1 (KM058087) from a soil sample [47]. Ariapiscaud viruses 1–5 (PQ490687-PQ490691), on the other hand, have very low genome-wide identity with other viruses (Supplementary Materials, Figure 3, Figure 4, Figure 5, Figure 6 and Figure 7) based on VIRIDIC [33] analyses.

The top BLASTn hits for the seven caudoviruses are summarized in the Supplementary Materials (Table S1). Wisapiscaud virus 1 (PQ490692) has the highest shared identity of 92.76% with 44% genome coverage compared to *Caudoviricetes* sp. 443 (MN855779), which was also identified in honeybee samples collected in Belgium [14]. Compared to the available sequences deposited in the NCBI GenBank, ariapiscaud virus 1–5 share 67–95% identity with 1–32% genome coverage (Supplementary Materials, Table S2).

Based on the ViPTree [35] analyses, the seven caudoviruses from this study are distributed across six different clades, and ariapiscaud virus 1 (PQ490687) is a divergent virus that groups distantly with other known caudoviruses (Figure 3). The results from VIRIDIC [33] analysis indicate that only wisapiscaud virus 1 (PQ490692) and wisapiscaud virus 2 (PQ490693) cluster with their closest neighbors Shigella virus Moo19 (MZ358387) and Cronobacter phage vB_CsaP_Ss1 (KM058087). Furthermore, only wisapiscaud virus 1 (PQ490692) clusters with viruses classified into a genus, i.e., *Schitoviridae* (Figure 2).

Host prediction using iPHoP [34] revealed that bacteria in the genus *Escherichia* are predicted as putative hosts for wisapiscaud virus 1, identified in honeybees housed at Marquette University in Wisconsin. Additionally, bacteria in the genus *Apilactobacillus* were predicted hosts for ariapiscaud virus 2 and those in the genus *Bombilactobacillus* for ariapiscaud virus 4, identified from honeybees on ASU Tempe campus. Lastly, bacteria in the genus *Serratia* were predicted as hosts for ariapiscaud virus 5, identified in honeybees from Tucson (Supplementary Materials, Table S3). The bacterial genera *Serratia*, *Escherichia*, *Bombilactobacillus*, and *Apilactobacillus* have all been previously identified in honeybees [48,49,50]. Ariapiscaud virus 4 has *Bombilactobacillus mellis* as the predicted host species Supplementary Materials, Table S3).

### 3.2. Inoviruses

Members of the family *Inoviridae* are flexible filamentous bacteriophages in the order *Tubulavirales*. Inoviruses have circular, positive-sense, single-stranded DNA genomes ranging from 5.5 to 10.5 kb. Currently, the family *Inoviridae* is comprised of 26 genera [51]. Members of the same genus share >50% amino acid sequence identity for the Zonula occludens toxin protein (ZOT) and major capsid protein, whereas members of the same species have >95% genome nucleotide sequence similarity [51].

Seven circular genomes encoding a ZOT protein and replication initiation protein were identified in this study (Figure 5). Additionally, they also encode a capsid protein, integrases, transposases, attachment proteins, and RstB-like proteins (Figure 6). These seven inoviruses have been named ariapisin virus 1–4 (name derived from **Ari**zona **Apis in**ovirus) and wisapisin virus 1 (name derived from **Wis**cosin **Apis in**ovirus) (Supplementary Materials, Table S1). The genomes of three ariapisin virus 1 (PQ490703, 7227 nt, PQ490704, 7227 nt, PQ490705, 7229 nt), from bees from three location in Arizona (ASU campus, Tempe Grand Canal, and Sedona) share >98.83% similarity. These three viruses, as well as ariapisin virus 2 (PQ490701, 8485 nt), ariapisin virus 3 (PQ490702, 7487 nt), ariapisin virus 4 (PQ490706, 5976 nt), and wisapisin virus 1 (PQ490707, 6268 nt), share very low genome-wide pairwise identity (Supplementary Materials, Figure S8), and their BLASTn analysis shows that they share 65–72.3% identity with 6–19% genome coverage to other inoviruses (Supplementary Materials, Table S2). The diverse nature of these inoviruses is also corroborated by the ViPTree proteomic phylogenetic tree, where they do not cluster closely with other known inoviruses (Figure 5).

Based on the iPHoP [34] host prediction, *Snodgrassella communis* is predicted as a putative host for ariapisin virus 1 (PQ490703-PQ490705), whereas bacteria in the genus *Gilliamella* are predicted for ariapisin virus 2 (PQ490701) and those in *Acinetobacter* are predicted for ariapisin virus 3 (PQ490702) and ariapisin virus 4 (PQ490706). No host was identified for wisapisin virus 1 (PQ490707). The bacterial genera and species predicted to be hosts of these viruses have all been previously identified in studies to be potential commensal members of honeybee microbiomes.

### 3.3. Microviruses

Microviruses have circular ssDNA genomes ranging in size from 3 to 6 kb [52,53], and their genomes are the smallest amongst the DNA phage groups [54]. Within microviruses, the major capsid protein and the replication initiator protein are the most well conserved [53]. The family *Microviridae* (order *Petitvirales*) has two subfamilies, *Gokushovirinae* and *Bullavirinae*, which are based on morphological and genomic characteristics such as virion structure and encoded scaffold proteins [52]. Additional subfamilies, including Alpavirinae [55], Amoyvirinae [56], Occultatumvirinae [57], Pichovirinae [58], Pequenovirinae [57], Reekeekeevirinae, Roodoodoovirinae [54], and Tainavirinae [57], have been unofficially proposed through the literature on microviruses. Currently, there are seven genera in the family *Microviridae* that are recognized by ICTV as of 2021 [46]. Within the subfamily *Bullavirinae*, there are three genera named *Alphatrevirus*, *Gequatrovirus,* and *Sinsheimervirus.* Within the subfamily *Gokushovirinae*, there are four genera named *Bdellomicrovirus*, *Chlamydiamicrovirus*, *Enterogokushovirus*, and *Spiromicrovirus.*

Two hundred eighty-eight microvirus genomes ranging in size from 4 to 6.1 kb were de novo assembled from honeybees (*n* = 286) and hoverflies (*n* = 2) in this study. The microvirus genomes assembled in this study share <95% genome-wide identity with each other. Of these 288 microviruses, a vast majority were from the ASU Tempe campus sample (sample ID HBAZ1; *n* = 155 microviruses), with 46 from the Tucson sample (sample ID # HBAZ1) and 34 from the White Tank Mountains sample ID # HBAZ6 (Table 1). Two of the microvirus genomes (PQ491799-PQ491800) are from the honeybee samples from Wisconsin (sample ID #HBWI) and have been named wisapismic virus 1–2, with the name being derived from **Wis**cosin **Apis mic**rovirus. Two microviruses (PQ491801-PQ491802) are from hoverflies from Rackensack Canyon (sample ID HFAZ), and these have been named arisyrphomic virus 1-2 (name derived from **Ari**zona **Syrpho**idea **mic**rovirus). The remaining 284 microvirus genomes (PQ491803-PQ492086), all from honeybee samples in Arizona, have been named ariapismic viruses 1–284 (derived from **Ari**zona **Apis mic**rovirus) (Supplementary Materials, Table S1).

Analysis of the microvirus genomes using BLASTn (Supplementary Materials, Table S2) revealed that ariapismic virus 160 (PQ491964) shares 100% identity with full genome coverage with wigfec virus K19 151 (OP549805), identified in the feces of an American wigeon (*Mareca americana*) [59] in Tempe, Arizona. Ariapismic virus 224 (PQ492052) shares 95.4% identity (99% genome coverage) with Microviridae sp. cthXX492 (MW202819) from a freshwater spring in Florida (USA) [60]. Other than this, all other microvirus genomes form this study share <87.1% identity with genome coverage between 1 and 88% (Supplementary Materials, Table S2). Seventeen of the microvirus genomes assembled in this study share the highest similarities (65.51–79.10% identity; 25–91% genome coverage) to previously identified microviruses from honeybees by Kraberger et al. [15], ranging from 65.51–79.10%. A further five microviruses had the highest similarity (67.53–74.56% identity; 23–72% genome coverage) to microviruses identified in blackflies (*Austrosimulium australense*) from New Zealand [61].

A proteomic phylogeny of the microviruses coupled with the vConTACT 2 analysis shows that microviruses from this study cluster together with those previously identified from insects (Figure 6). In general, a large portion of the microviruses identified in this study are part of the *Gokushovirinae* subfamily (honeybees from this study, *n* = 156; hoverflies from this study, *n* = 2) (Supplementary Materials, Table S1). Furthermore, in this subfamily grouping, we also note that microviruses were identified in honeybees from a prior study (*n* = 45) [15], as well as blackflies (*n* = 92) [61]. Group D ispredominantly composed of microviruses from invertebrates and is the second largest cluster in which the microviruses from this study, along with microviruses of other honeybee/insect studies, group into. This grouping has 90 genomes from this study (Supplementary Materials, Table S1), 18 from honeybees from the study by Kraberger et al. (2019) [15], and two from blackflies from the study by Kraberger et al. (2019) [61]. The remaining 40 microvirus genomes are part of the groupings Alpavirinae C3 (*n* = 3), Occultatum/Tainovirinae (*n* = 1), Pichovirinae (*n* = 25), Pequenovirinae (*n* = 3), VC1 (*n* = 7), and VC4 (*n* = 1) (Supplementary Materials, Table S1).

A broader analysis of the microviruses from insects from this study coupled with two other studies that have generated microvirus sequences from honeybees [15] and blackflies [61] (Figure 7) shows that 54.5–64.3% of the microvirus genomes from bees are part of the *Gokushovirinae* subfamily. In the case of blackflies, this is 85.2%. Furthermore, in honeybees, 25.7–31.5% of the microvirus genomes are part of Group D and 8.6–8.7% are part of the Pichovirinae group, compared to 1.9% and 11.1% for blackflies, respectively (Figure 7).

Using iPHoP [34] host prediction, we were able to identify putative hosts for 22 microviruses from this study. Nineteen of these microviruses (ariapismic virus 12, 15, 35, 43, 60, 62, 63, 86, 134, 161, 162, 174, 193, 206, 231, 238, 256, 262, 283; PQ491814, PQ491817, PQ491837, PQ491845, PQ491862, PQ491864, PQ491865, PQ491888, PQ491936, PQ491965, PQ491966, PQ491977, PQ491992, PQ492013, PQ492020, PQ492034, PQ492057, PQ492063, PQ492084), which all are part of the *Gokushovirinae* subfamily, have predicted hosts in the genus *Phascolarctobacterium*. Bacteria of the genus *Phascolarctobacterium* have primarily been identified in blood, intestinal, and fecal samples originating from humans and other animals. One exception is a sequence of a bacterium within this genus that has been identified from Asian honeybees of the Indian subcontinent [62]. Since bacteria in the genus *Phascolarctobacterium* have predominantly been identified in animal and environmental samples, it is possible that these host bacteria are members of the transient community of bacteria acquired during foraging and contact with animal fecal samples. Wisapismic virus 1 (PQ491799) is part of the VC4 cluster and has a member of *Prevotella* [63] as a putative host. Ariapismic virus 74 (PQ491876), in *Gokushovirinae*, is predicted to be hosted by bacteria in the genus *Duodenibacillus*. Ariapismic virus 227 (PQ492009), also in *Gokushovirinae,* is predicted to be hosted by bacteria in the genus/genera *Chlamydophila*/*Chlamydia* (Supplementary Materials, Table S3). While bacteria of the genus *Prevotella* have also been identified in sampled bees, the association between bacteria of the genera *Duodenibacillus* and *Chlamydia* with honeybees is less represented.

### 3.4. Distribution of Bacteriophages

**Figure 8:**
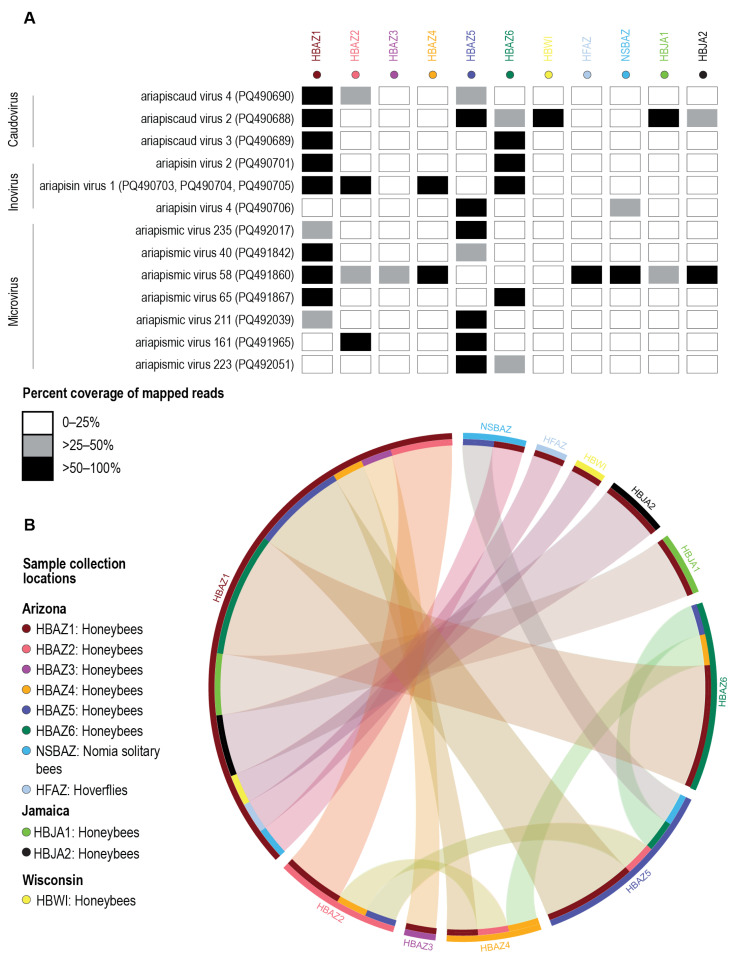
(**A**) Summary of presence/absence matrix of caudovirus, inovirus, and microvirus sequences across all samples when present in at least two sample pools. Matrix for all viruses is provided in Supplementary Materials, Figure S9. Viruses were considered present in a sample pool if at least 25% of the genome had read coverage. (**B**) A circo-plot displaying the connection to sample pools that showed the presence of the virus in sample pools where the virus was first identified is displayed at the bottom of the read mapping array. Sample location identities and color schemes are displayed according to the legend on the bottom left.

To determine the distribution of virus sequences (but incomplete de novo assembled genomes) across all the samples, we mapped the raw reads to 300 of the 302 unique viruses (vOTU threshold of 98%). When mapping the raw reads, we used a 25% mapping genome coverage as a positive indication that the bacteriophage was present in the pooled sample (Supplementary Materials, Figure S9). Many of the virus vOTUs were only identified in a single sample; however, for 13, we identified these in multiple samples, and we have summarized these in detail in Figure 8.

Ariapiscaud virus 2 (PQ490688), whose predicted hosts are in the genus *Apilactobacillus* (Supplementary Materials, Table S3), was present in six of the 11 pooled samples, including honeybees from the ASU Tempe campus (sample ID # HBAZ1), Tucson (sample ID # HBAZ5), the White Tank Mountains (sample ID # HBAZ6), Wisconsin (sample ID # HBWI), and both apiaries in Jamaica (sample ID # HBJA1, HBJA2) (Figure 8). Ariapiscaud virus 4 (PQ490690), whose predicted host is *Bombilactobacillus mellis* (Supplementary Materials, Table S3), was present in samples from the ASU Tempe campus (sample ID # HBAZ1), Tempe Grand Canal (sample ID # HBAZ2), and Tucson (sample ID # HBAZ5), whereas ariapiscaud virus 3 (PQ490689) was present in bees from the ASU Tempe campus (sample ID # HBAZ1) and the White Tank Mountains (sample ID # HBAZ6). These two bacteriophages are found in three and two pooled samples, respectively (Figure 8).

Genome sequences of ariapisin virus 1 (PQ490703, PQ490704, PQ490705), whose predicted host is *Snodgrasella communis*, were de novo assembled from bees on the ASU Tempe campus (sample ID # HBAZ1), Tempe Grand Canal (sample ID # HBAZ2), and in Sedona (sample ID # HBAZ4) and present in the sample from the White Tank Mountains (sample ID # HBAZ6), all in Arizona. It is important to note that Sedona is ~200 km north of Tempe, whereas the White Tank Mountains are ~65 km west of Tempe (Figure 1), showing that this inovirus is present in bees over a greater spatial range. Ariapisin virus 2, whose predicted hosts are bacteria in the genus *Gilliamella*, was present in samples from the ASU Tempe campus (sample ID # HBAZ1) and the White Tank Mountains (sample ID # HBAZ6). Ariapisin virus 4, whose predicted hosts are bacteria in the genus *Acinetobacter*, was found in samples from Tucson (sample ID # HBAZ5) and present in *Nomia* solitary bees from the Tempe Grand Canal (sample ID # NSBAZ), suggesting a broader distribution of its host across insect taxa (Figure 8).

Ariapismic virus 58 (PQ491860) in the VC1 group (Supplementary Materials, Table S1, Figure 6) was present seven pooled samples, i.e., the ASU Tempe campus (sample ID # HBAZ1), Tempe Grand Canal (sample ID # HBAZ2), Rackensack Canyon honeybees (sample ID # HBAZ3), Sedona (sample ID # HBAZ4), both apiaries in Jamaica (sample ID # HBJA1, HBJA2), *Nomia* solitary bees from the Tempe Grand Canal (sample ID # NSBAZ), and hoverflies from Rackensack Canyon (sample ID # HFAZ). Ariapismic virus 58 is the most identified bacteriophage in this study, having the broadest spatial range and distribution across insect taxa (Figure 1 and Figure 8). Ariapismic virus 40 (PQ491842), ariapismic virus 65 (PQ491867), ariapismic virus 211 (PQ492039), ariapismic virus 161 (PQ491965), and ariapismic virus 223 (PQ492051), all in the subfamily *Gokushovirinae*, were all present in at least two honey bee samples collected in bees within a ~60 km radius of the ASU Tempe campus (Figure 8), whereas ariapismic virus 235 (PQ492017) in the subfamily *Gokushovirinae* was present in samples collected at the ASU Tempe campus (sample ID # HBAZ1) and in Tucson (sample ID # HBAZ5) with a wider spatial distribution over ~200 km.

In general, our results on the distribution of bacteriophages corroborate the findings of Busby et al. (2022) [13], who showed that a majority of phages associated with the honeybee microbiome are more specific to the local areas where they are sampled.

## 4. Conclusions

In this study, we identified 302 complete phage genomes from honeybees (*n* = 300) and hoverflies (*n* = 2) sampled in Arizona and Wisconsin in the USA, as well as from honeybees sampled in Jamaica (Supplementary Materials, Table S1). Although no full genomes were assembled from the Nomia bees, read mapping showed the presence of a microvirus and an inovirus. From a total of eight pooled honeybee samples, 300 genomes represent new species of caudoviruses (*n* = 7), inoviruses (*n* = 5), and microviruses (*n* = 286). These viruses meet the 95% species demarcation threshold for bacteriophages [46] as they all share <95% genome-wide pairwise identity with other related bacteriophages. Several phages (caudovirus *n* = 3, inovirus *n* = 3, microvirus *n* = 7) were present in two or more samples of bees and hoverflies from more than one location or sample type.

The hosts of the caudoviruses and inoviruses predicted with iPHoP [34] are consistent with bacteria that have previously been identified in honeybee microbiomes. Amongst the seven identified caudoviruses, host bacteria were identified to the genus level with confidence for four phages. The predicted hosts were observed from bacterial genera that have previously been identified to be present in host microbiomes, including *Apilactobacillus*, *Bombilactobacillus*, *Escherichia*, and *Serratia*. Amongst the five inoviruses identified in this study, bacterial hosts were predicted for four phages. For the 288 microviruses identified in this study, hosts for only 22 microviruses were predicted. Of these 22 bacteriophages, 19 are likely associated with human and animal bacteria in the genus *Phascolarctobacterium.* The remaining ones have putative predicted bacteria hosts in the genera *Duodenobacillus*, *Chlamydia*, and *Prevotella*. For the microviruses, the predicted bacterial hosts have not been identified frequently in studies of the honeybee microbiome. It is likely that microviruses are infecting more transient bacteria communities in honeybees.

This study aimed to identify bacteriophage genomes in bees and hoverflies. While this study focuses primarily on the bacteriophages associated with the microbiome of honeybees, we sought to address the lack of information associated with this topic and other insects by including information gathered from opportunistically sampled solitary bees and hoverflies.

By identifying, characterizing, and understanding the lineages of these bacteriophages, this study provides insights into the prokaryotic virome of several pollinating insects and significantly expands the total number of prokaryotic virus genomes associated with bees and hoverflies.

## Figures and Tables

**Table 1:** Summary of the number of full bacteriophage genome groupings (*Caudoviricetes*, *Inoviridae*, and *Microviridae*) de novo assembled from each sequencing pool. The location, sequencing pool names, and number of sampled organisms are also provided.

Sample Location	Sequencing Pool Name	Sample Type (Number of Individuals Collected)	Caudovirus Genomes	Inovirus Genomes	Microvirus Genomes	Total Bacteriophage Genomes
ASU Tempe campus	HBAZ1	Honeybees (*n* = 20)	4	3	155	162
Tempe Grand Canal	HBAZ2	Honeybees (*n* = 20)	0	1	10	11
Tempe Grand Canal	NSBAZ	Nomia solitary bees (*n* = 20)	0	0	0	0
Jamaica—site 1	HBJA1	Honeybees (*n* = 20)	0	0	0	0
Jamaica—site 2	HBJA2	Honeybees (*n* = 20)	0	0	0	0
Rackensack Canyon	HBAZ3	Honeybees (*n* = 16)	0	0	34	34
Rackensack Canyon	HFAZ	Hoverflies (*n* = 15)	0	0	2	2
Sedona	HBAZ4	Honeybees (*n* = 20)	0	1	5	6
Tucson	HBAZ5	Honeybees (*n* = 20)	1	1	46	48
Wisconsin	HBWI	Honeybees (*n* = 20)	2	1	2	5
White Tank Mountains	HBAZ6	Honeybees (*n* = 20)	0	0	34	34

## Data Availability

Raw reads have been deposited in NCBI SRA under BioProject PRJNA1171949. Biosample and SRA accessions are as follows: HBAZ1 (Arizona honeybees—ASU Tempe campus): BioSample # SAMN44294404, SRA # SRR31054156; BioSample # SAMN44294403, SRA #SRR31054157; BioSample # SAMN44260432, SRA #SRR30976612. HBAZ2 (Arizona honeybees—Tempe grand canal): BioSample # SAMN44338419, SRA #SRR31054581; BioSample # SAMN44260436, SRA #SRR30976607. HBAZ3 (Arizona honeybees—Rackensack canyon): BioSample # SAMN44260437, SRA #SRR30976606. HBAZ4 (Arizona honeybees—Sedona): BioSample # SAMN44260438, SRA #SRR30976605. HBAZ5 (Arizona honeybees—Tucson): BioSample # SAMN44260439, SRA #SRR30976604. HBAZ6 (Arizona honeybees—White Tank mountains): BioSample # SAMN44260441, SRA #SRR30976602. HBWI (Wisconsin honeybees): BioSample # SAMN44260440, SRA #SRR30976603. HFAZ (Arizona hoverflies—Rackensack canyon): BioSample # SAMN44260442, SRA #SRR30976610. Assembled genomes have been deposited in NCBI GenBank under accession # PQ490687-PQ490693 for caudoviruses, PQ490701-PQ490707 for inoviruses, and PQ491799-PQ492086 for microviruses.

## Supplementary Materials

The following supporting information can be downloaded at: https://www.mdpi.com/article/10.3390/v17020201/s1, Figure S1: VIRIDIC intergenomic identity plot of a subset of the caudovirus phylogeny that includes wisapiscaud virus 1 (PQ490692) from Figure 2; Figure S2: VIRIDIC intergenomic identity plot of a subset of the caudovirus phylogeny that includes wisapiscaud virus 2 (PQ490693) from Figure 2; Figure S3: VIRIDIC intergenomic identity plot of a subset of the caudovirus phylogeny that includes ariapiscaud virus 1 (PQ490687) from Figure 3; Figure S4: VIRIDIC intergenomic identity plot of a subset of the caudovirus phylogeny that includes ariapiscaud virus 2 (PQ490688) from Figure 3; Figure S5: VIRIDIC intergenomic identity plot of a subset of the caudovirus phylogeny that includes ariapiscaud virus 3 (PQ490689) from Figure 3; Figure S6: VIRIDIC intergenomic identity plot of a subset of the caudovirus phylogeny that includes ariapiscaud virus 4 (PQ490690) from Figure 4; Figure S7: VIRIDIC intergenomic identity plot of a subset of the caudovirus phylogeny that includes ariapiscaud virus 5 (PQ490691) from Figure 4; Figure S8: VIRIDIC intergenomic identity plot of invorises including wisapisin virus 1 (PQ490707), ariapisin virus 1 (PQ490703-PQ490705), ariapisin virus 2 (PQ490701), ariapisin virus 4 (PQ490706), ariapisin virus 3 (PQ490702) from Figure 5; Figure S9: Summary of presence/absence of the viruses identified in this study across the various samples. The presence/absence is determined based on read mapping to the full genomes using BBMap and the percentage genome coverage is provided for each virus with >25% being considered as positive; Table S1: Summary of the bacteriophages identified in this study with Class, Order, Family and group details based on VIPTree and vConTACT 2 analysis. The GenBank accession numbers and corresponding BioProject, BioSample and SRA accession numbers are provided; Table S2: Summary of the NCBI Blastn results of the bacteriophage genomes identified in this study showing the top hit with query coverage, percentage identity and E-value; Table S3: Summary of the host prediction with iPHoP for the bacteriophage genomes identified in this study.
