# Supplementary material for: Identification of Diverse Bacteriophages Associated with Bees and Hoverflies
Source: Viruses. 2025 Jan 30;17(2):201. doi: 10.3390/v17020201 (PMC11860568; doi:10.3390/v17020201)
Supplement: Supplementary file 1 [file viruses-17-00201-s001.zip › Proof sup figures/Supplementary figure 2_v5.pdf]

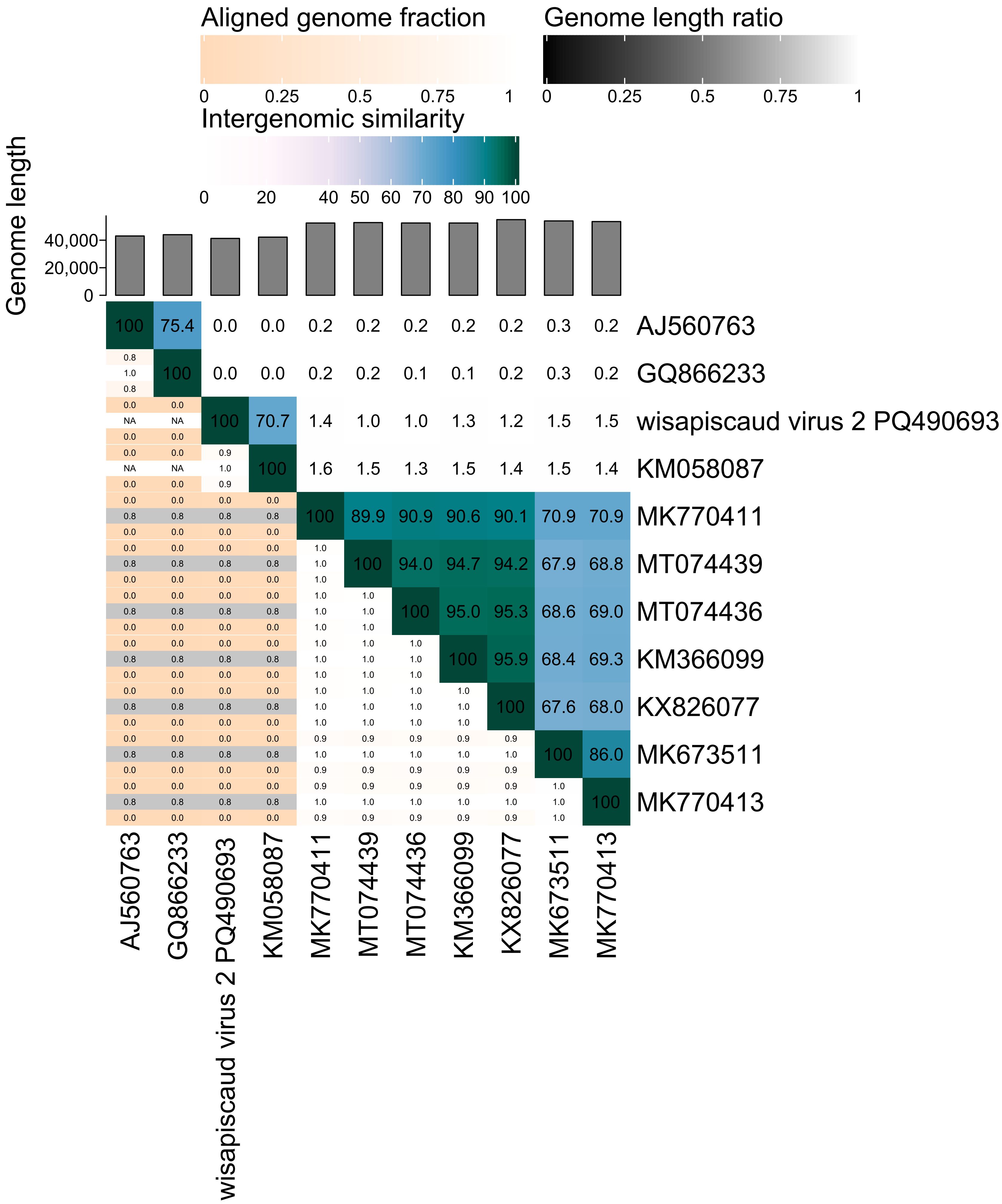

Aligned genome fraction
0
0.25
0.5
0.75
1
Genome length ratio
0
0.25
0.5
0.75
1
Intergenomic similarity
0
20
40
50
60
70
80
90
100
Genome length
40,000
20,000
0
AJ560763: 100 75.4 0.0 0.0 0.2 0.2 0.2 0.2 0.2 0.3 0.2
GQ866233: 100 0.0 0.0 0.2 0.2 0.1 0.1 0.2 0.3 0.2
wisapiscaud virus 2 PQ490693: 100 70.7 1.4 1.0 1.0 1.3 1.2 1.5 1.5
KM058087: 100 1.6 1.5 1.3 1.5 1.4 1.5 1.4
MK770411: 100 89.9 90.9 90.6 90.1 70.9 70.9
MT074439: 100 94.0 94.7 94.2 67.9 68.8
MT074436: 100 95.0 95.3 68.6 69.0
KM366099: 100 95.9 68.4 69.3
KX826077: 100 67.6 68.0
MK673511: 100 86.0
MK770413: 100
AJ560763
GQ866233
wisapiscaud virus 2 PQ490693
KM058087
MK770411
MT074439
MT074436
KM366099
KX826077
MK673511
MK770413
