# Supplementary material for: Identification of Diverse Bacteriophages Associated with Bees and Hoverflies
Source: Viruses. 2025 Jan 30;17(2):201. doi: 10.3390/v17020201 (PMC11860568; doi:10.3390/v17020201)
Supplement: Supplementary file 1 [file viruses-17-00201-s001.zip › Proof sup figures/Supplementary figure 5_v5.pdf]

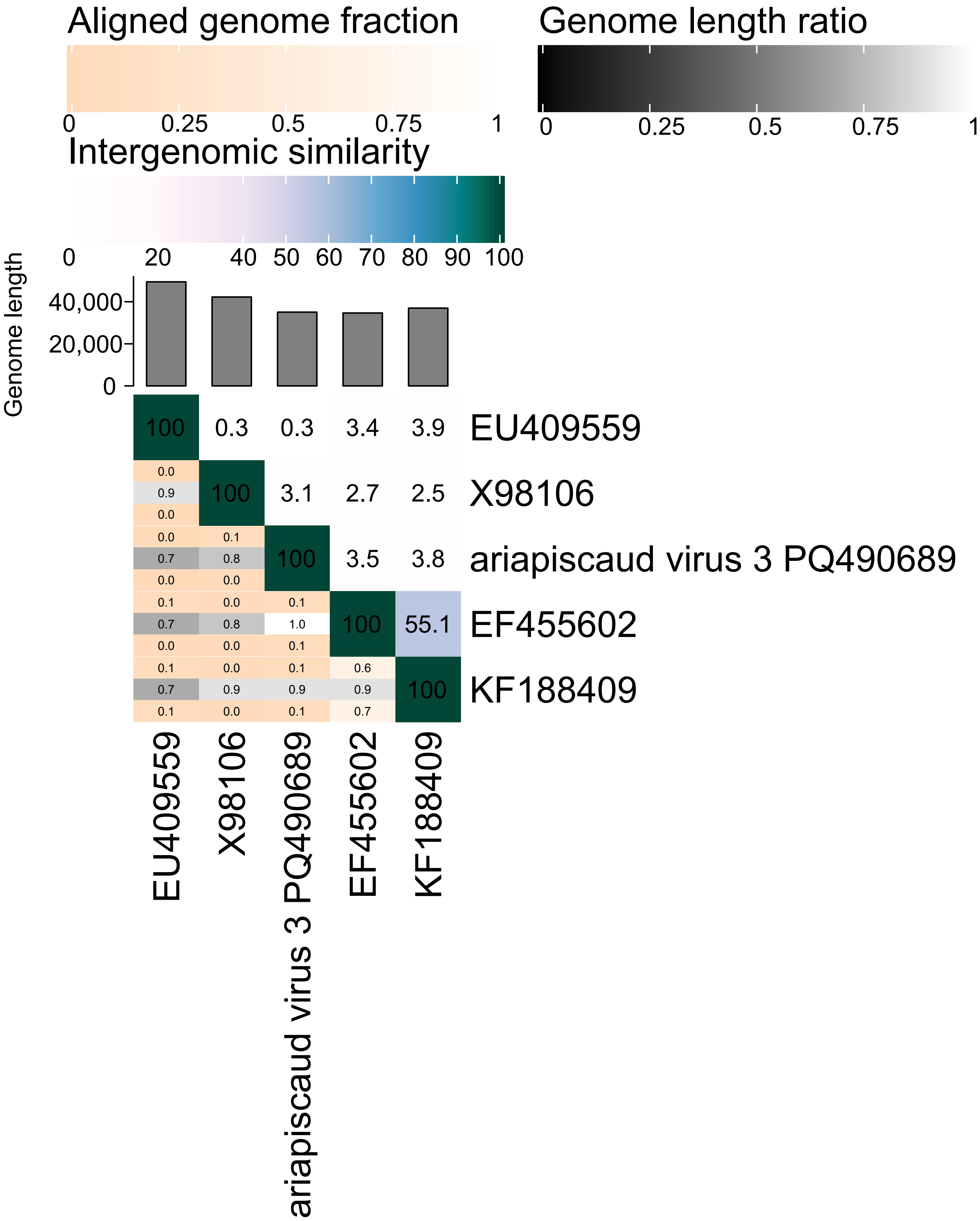
Aligned genome fraction
0
0.25
0.5
0.75
1
Genome length ratio
0
0.25
0.5
0.75
1
Intergenomic similarity
0
20
40
50
60
70
80
90
100
Genome length
40,000
20,000
0
EU409559
X98106
ariapiscaud virus 3 PQ490689
EF455602
KF188409
100
0.3
0.3
3.4
3.9
100
3.1
2.7
2.5
100
3.5
3.8
100
55.1
100
