# Supplementary material for: Identification of Diverse Bacteriophages Associated with Bees and Hoverflies
Source: Viruses. 2025 Jan 30;17(2):201. doi: 10.3390/v17020201 (PMC11860568; doi:10.3390/v17020201)
Supplement: Supplementary file 1 [file viruses-17-00201-s001.zip › Proof sup figures/Supplementary figure 6_v5.pdf]

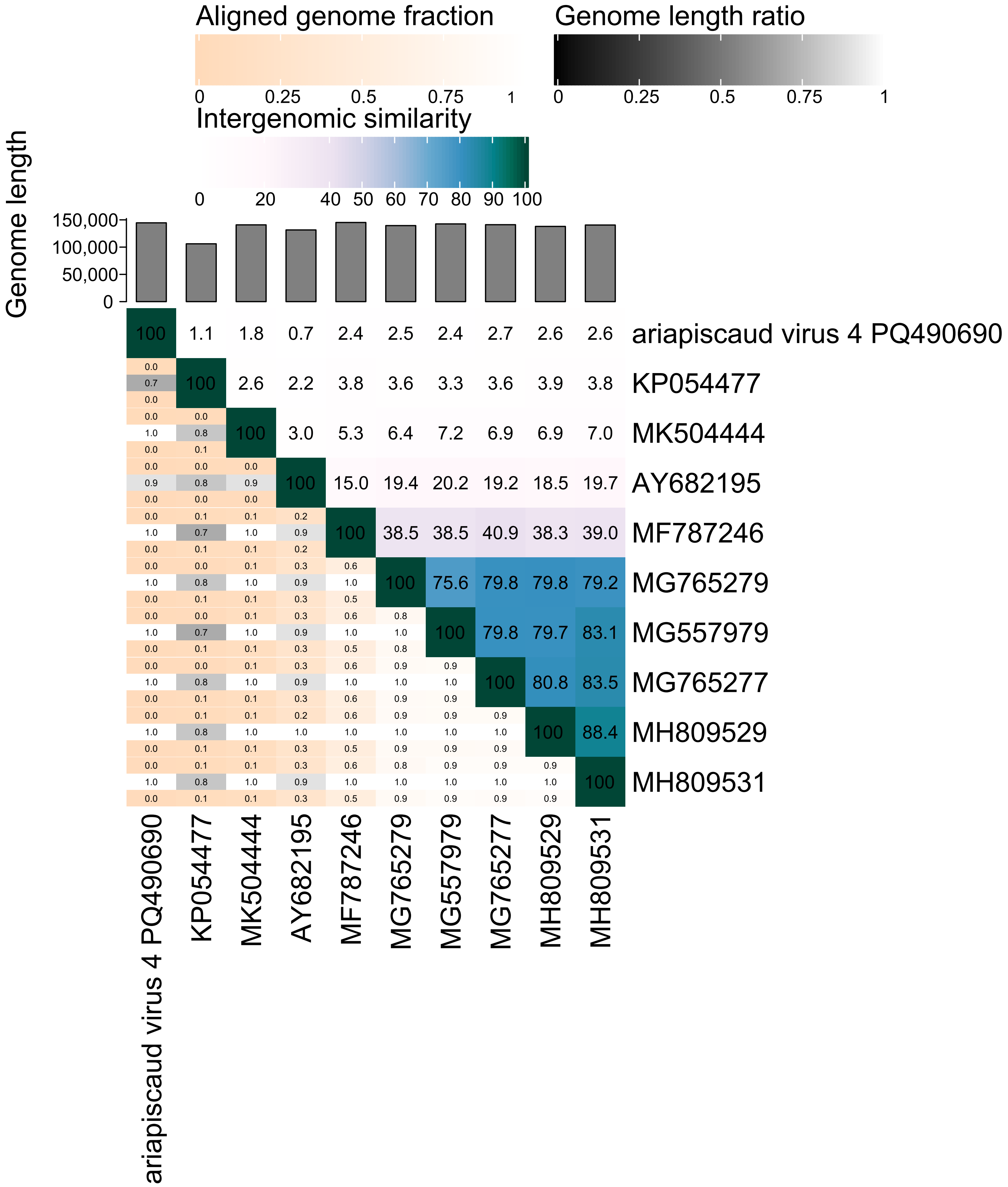

Aligned genome fraction
0 0.25 0.5 0.75 1
Genome length ratio
0 0.25 0.5 0.75 1
Intergenomic similarity
0 20 40 50 60 70 80 90 100
Genome length
150,000 100,000 50,000 0
100 1.1 1.8 0.7 2.4 2.5 2.4 2.7 2.6 2.6 ariapiscaud virus 4 PQ490690
100 2.6 2.2 3.8 3.6 3.3 3.6 3.9 3.8 KP054477
100 3.0 5.3 6.4 7.2 6.9 6.9 7.0 MK504444
100 15.0 19.4 20.2 19.2 18.5 19.7 AY682195
100 38.5 38.5 40.9 38.3 39.0 MF787246
100 75.6 79.8 79.8 79.2 MG765279
100 79.8 79.7 83.1 MG557979
100 80.8 83.5 MG765277
100 88.4 MH809529
100 MH809531
ariapiscaud virus 4 PQ490690
KP054477
MK504444
AY682195
MF787246
MG765279
MG557979
MG765277
MH809529
MH809531
