# Supplementary figures and images for: Identification of Diverse Bacteriophages Associated with Bees and Hoverflies
Source: Viruses. 2025 Jan 30;17(2):201. doi: 10.3390/v17020201 (PMC11860568; doi:10.3390/v17020201)

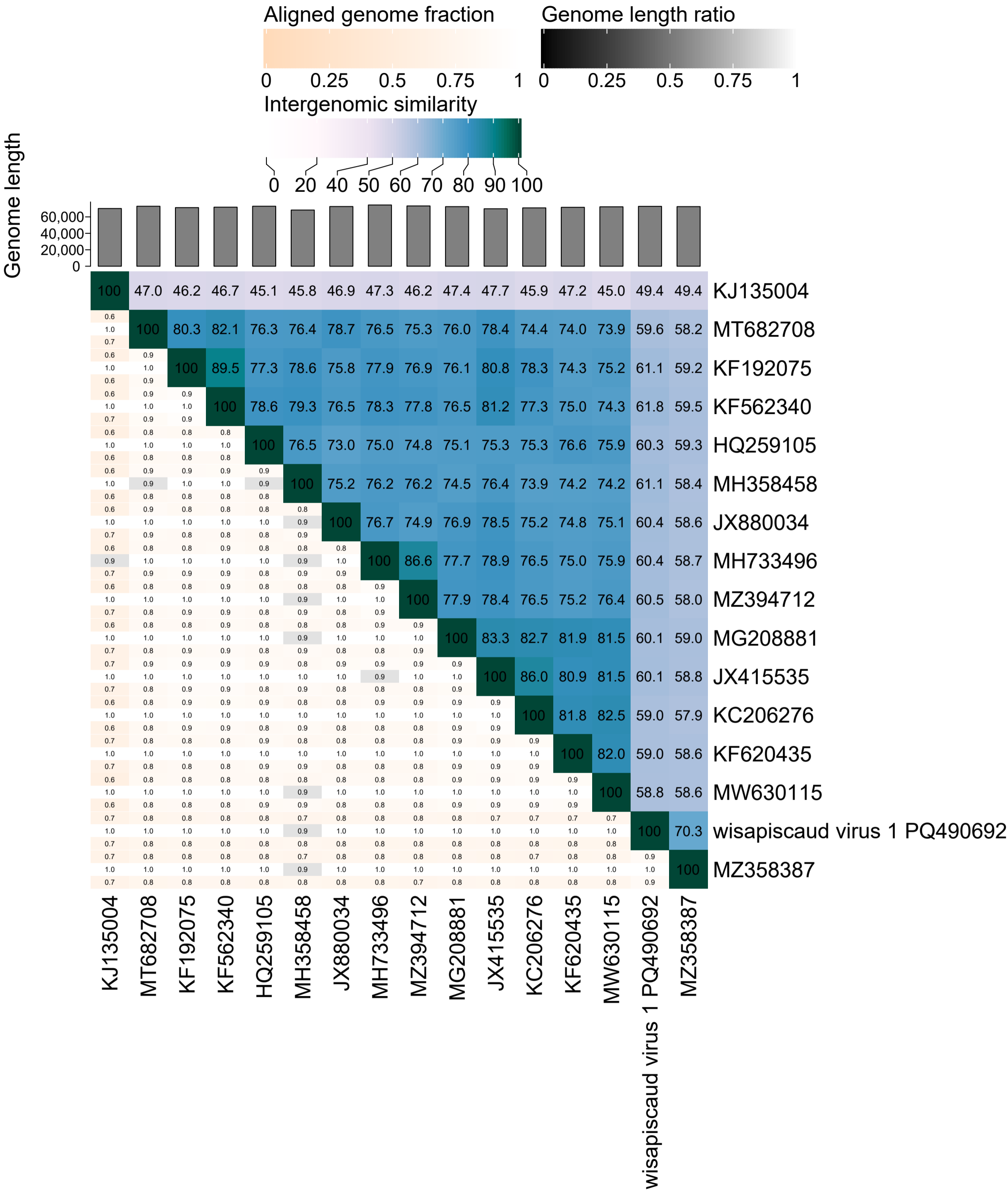

Supplement: Supplementary file 1 [file viruses-17-00201-s001.zip › Proof sup figures/Supplementary figure 1_v5.pdf]

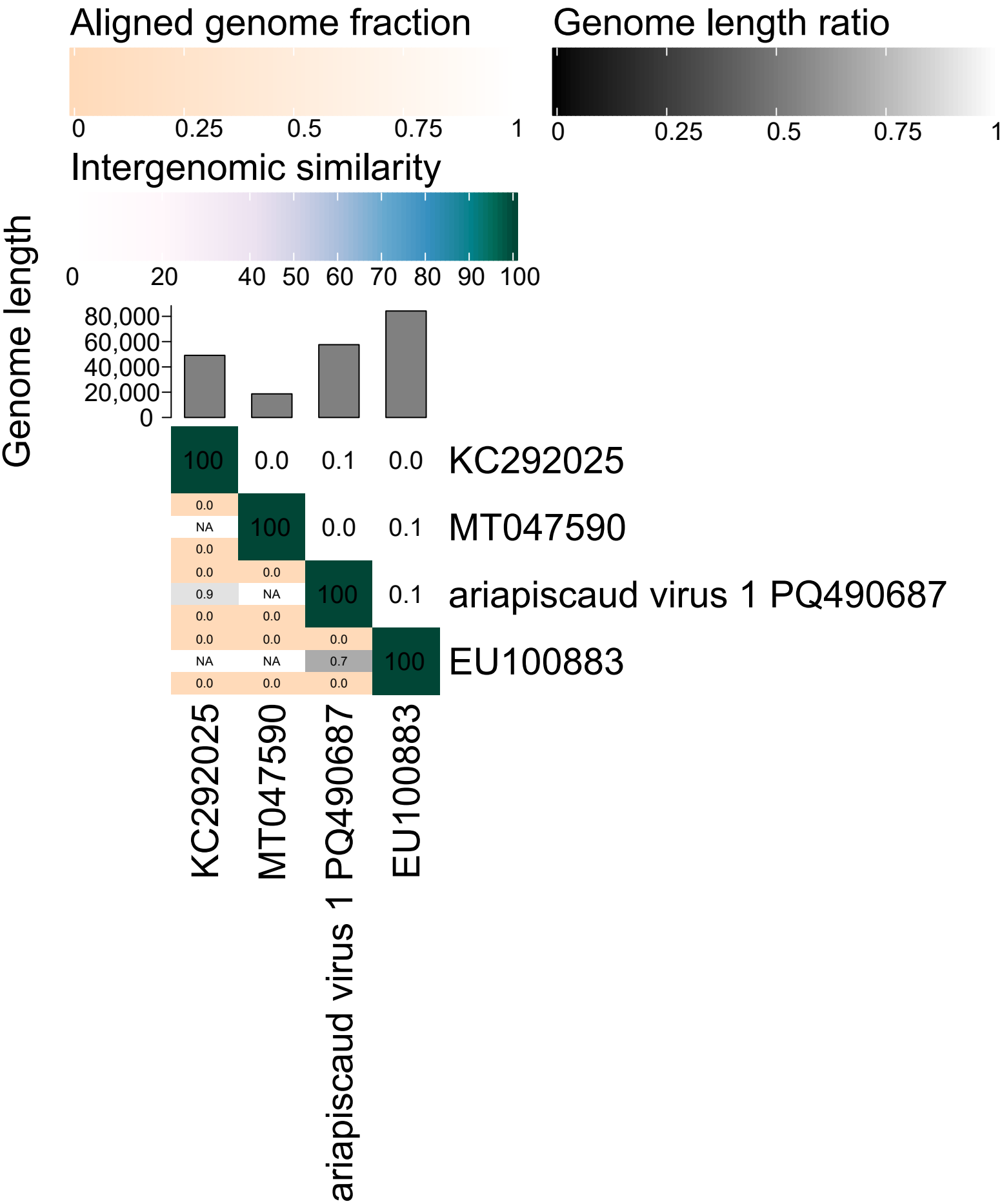

Supplement: Supplementary file 1 [file viruses-17-00201-s001.zip › Proof sup figures/Supplementary figure 3_v5.pdf]

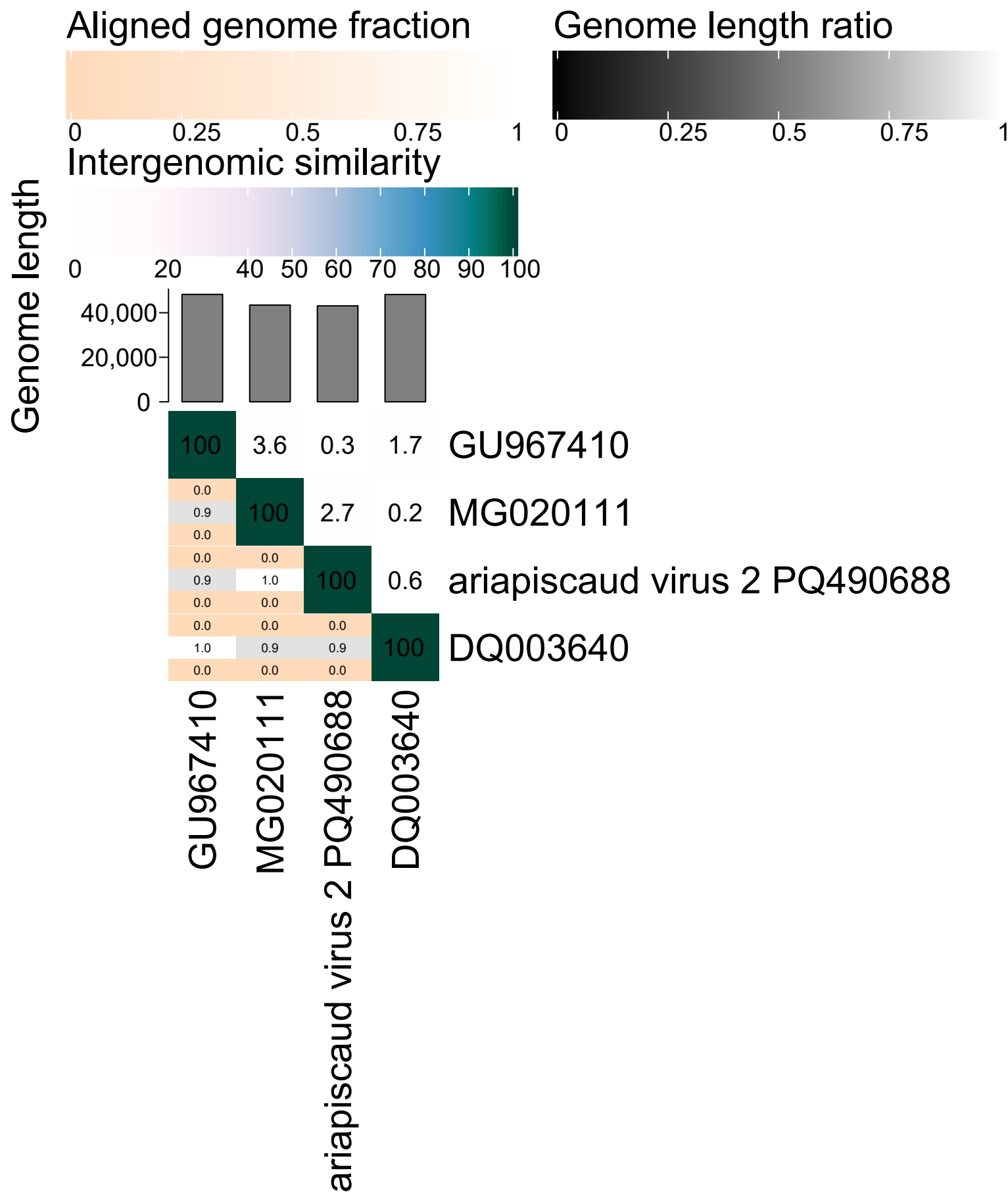

Supplement: Supplementary file 1 [file viruses-17-00201-s001.zip › Proof sup figures/Supplementary figure 4_v5.pdf]

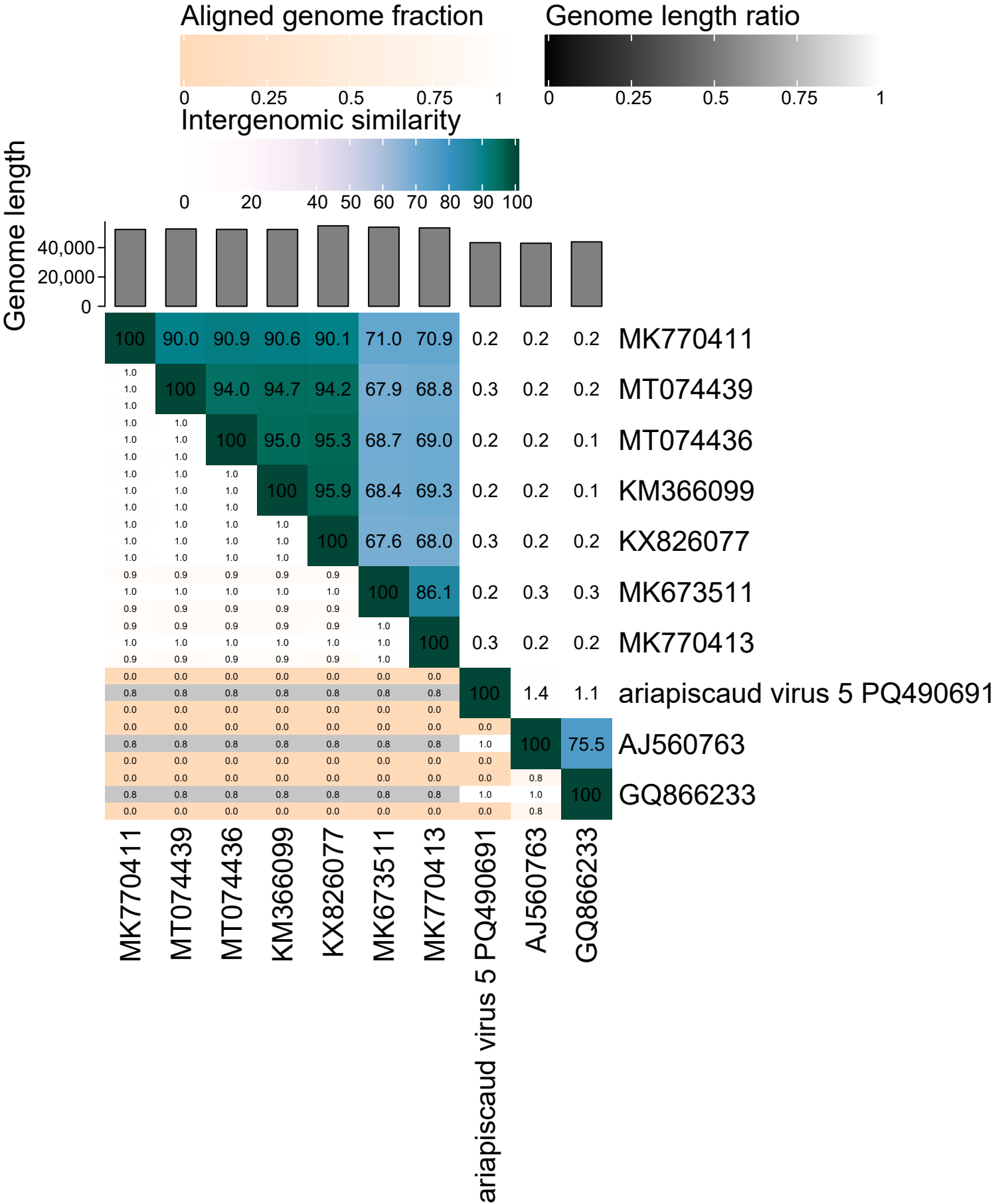

Supplement: Supplementary file 1 [file viruses-17-00201-s001.zip › Proof sup figures/Supplementary figure 7_v5.pdf]

Aligned genome fraction

Genome length ratio

0 0.25 0.5 0.75 10 0.25 0.5 0.75 1

Intergenomic similarity

0 20 40 50 60 70 80 90 100

Genome\_length

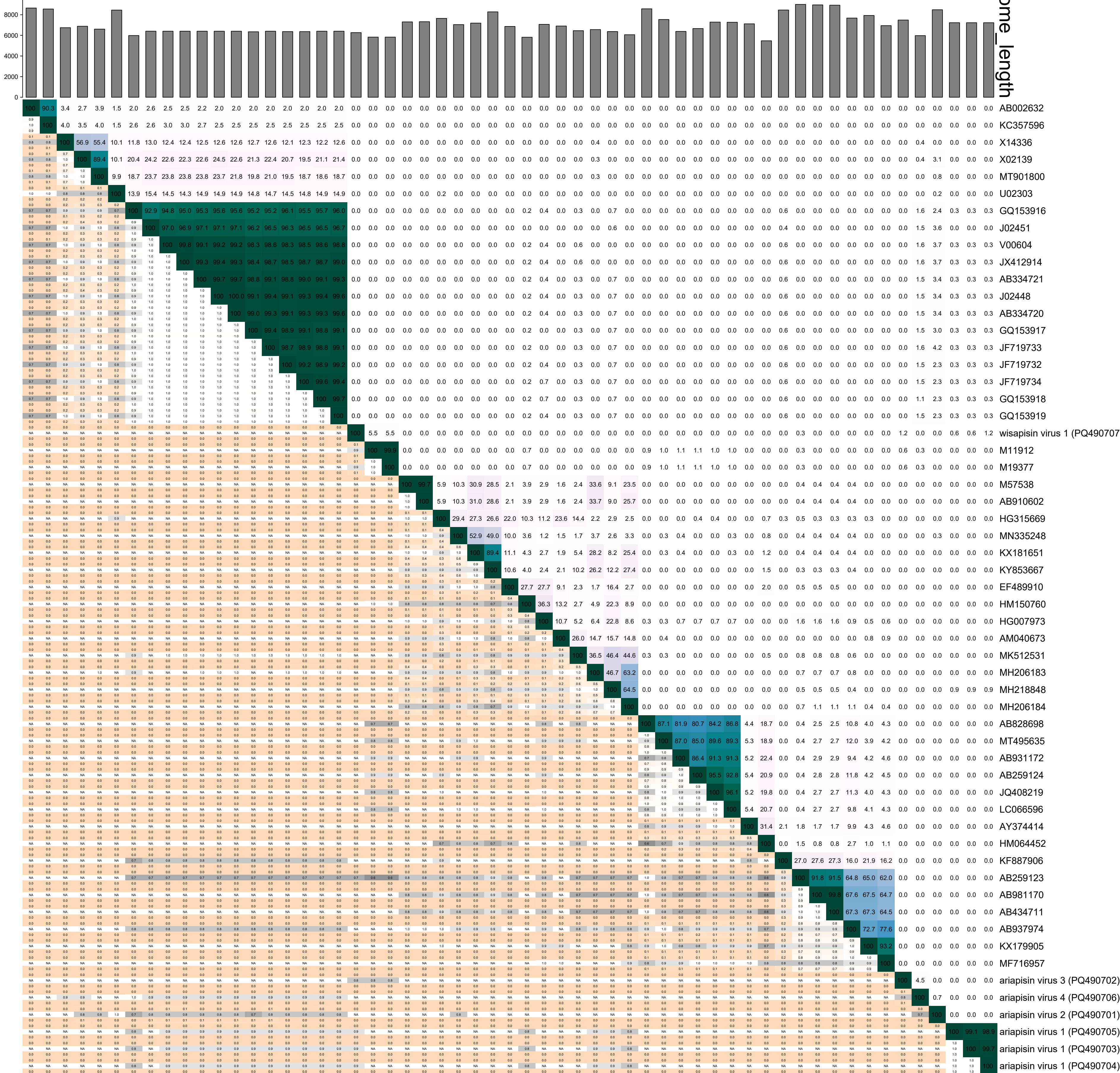

Supplement: Supplementary file 1 [file viruses-17-00201-s001.zip › Proof sup figures/Supplementary figure_8_v4.pdf]

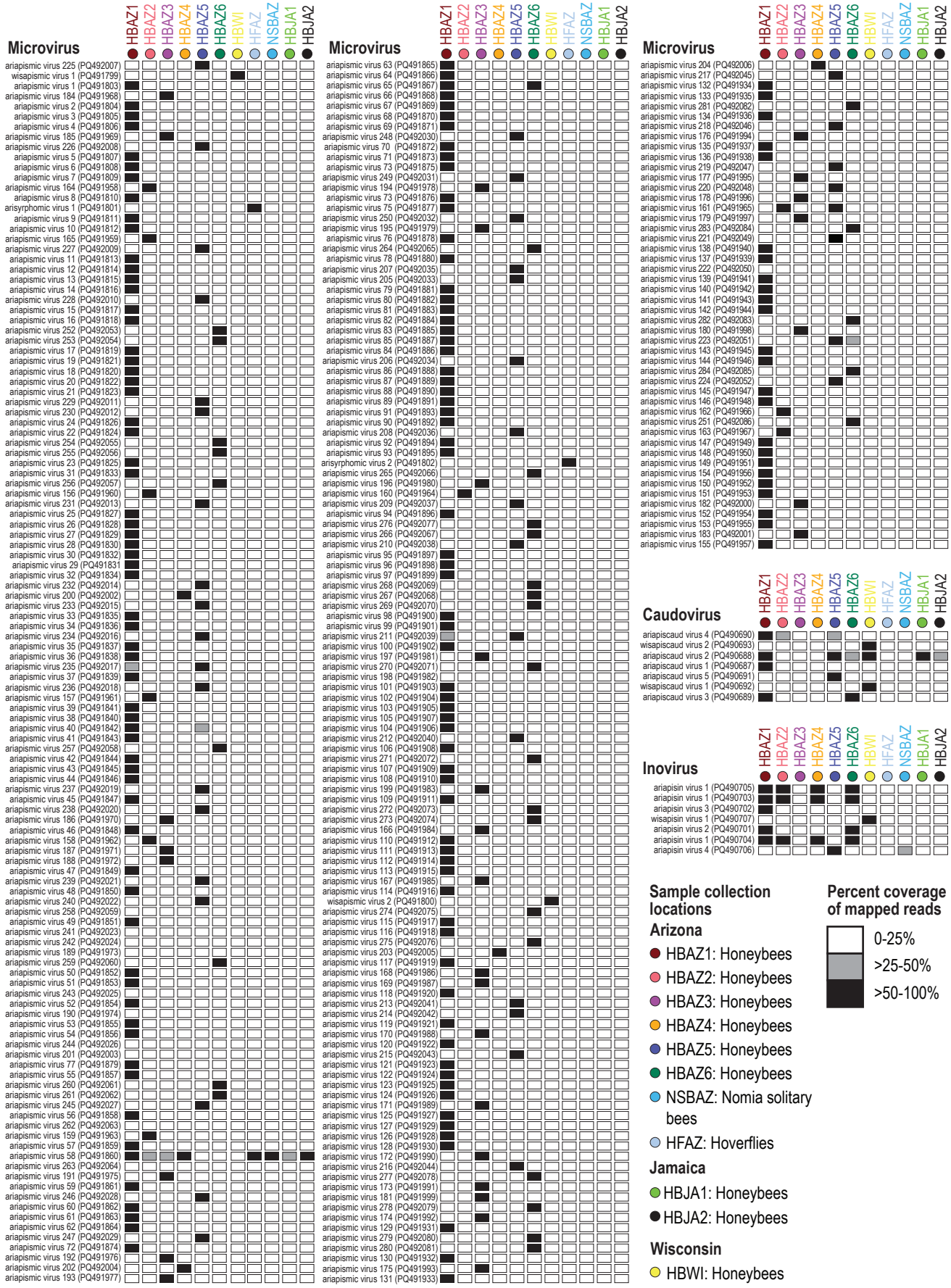

Supplement: Supplementary file 1 [file viruses-17-00201-s001.zip › Proof sup figures/Supplementary figure_9_v4.pdf]
